# Gasdermin E (GSDME)—A New Potential Marker of Psoriasis and Its Metabolic Complications: The First Combined Study on Human Serum, Urine and Tissue

**DOI:** 10.3390/cells12172149

**Published:** 2023-08-26

**Authors:** Julia Nowowiejska, Anna Baran, Anna Pryczynicz, Justyna Magdalena Hermanowicz, Beata Sieklucka, Dariusz Pawlak, Iwona Flisiak

**Affiliations:** 1 Department of Dermatology and Venereology, Medical University of Bialystok, 14 Zurawia St., 15-540 Bialystok, Poland; anna.baran@umb.edu.pl (A.B.); iwona.flisiak@umb.edu.pl (I.F.); 2 Department of General Pathomorphology, Medical University of Bialystok, 13 Waszyngtona St., 15-269 Bialystok, Poland; anna.pryczynicz@umb.edu.pl; 3 Department of Pharmacodynamics, Medical University of Bialystok, 2C Mickiewicza St., 15-089 Bialystok, Poland; justyna.hermanowicz@umb.edu.pl (J.M.H.); beata.sieklucka@umb.edu.pl (B.S.); dariusz.pawlak@umb.edu.pl (D.P.)

**Keywords:** GSDME, gasdermin, gasdermin E, pyroptosis, apoptosis, inflammation, cell proliferation

## Abstract

Psoriasis is a frequent and incurable skin disease whose pathogenesis is still not fully understood. It is characterized by immune disturbances leading to hyperproliferation and improper differentiation of keratinocytes. Gasdermin E (GSDME) is a protein from the gasdermin family involved in the processes of inflammation and cell death based on apoptosis, necroptosis and pyroptosis. It has never been studied in psoriatics’ sera or urine before. Our study enrolled 60 patients with psoriasis and 30 volunteers without dermatoses as controls. Serum and urinary GSDME concentrations were examined by ELISA and tissue expression of GSDME by immunohistochemistry. Serum GSDME concentration was significantly higher in patients than controls (*p* < 0.05). There were no differences in urinary GSDME concentrations between patients and controls. GSDME expression was significantly higher in the psoriatic plaque than non-lesional patients’ skin and compared to controls (both *p* < 0.001). There was no correlation between serum GSDME or its lesional expression and psoriasis severity, age or disease duration. GSDME serum concentration was significantly negatively correlated with BMI, triglycerides and glucose concentrations. The obtained results suggest the engagement of GSDME in psoriasis pathogenesis. It could potentially become a new non-invasive psoriasis marker. Considering its pro-apoptotic influence, GSDME could be compensatively elevated to direct cells towards apoptosis, whereas under other circumstances, it may lead to pyroptosis and sustain inflammation. GSDME may exert a protective influence on the metabolic complications in psoriasis which requires further studies.

## 1. Introduction

Psoriasis is a frequent, chronic and incurable skin disease that occurs with a prevalence of 2–3% in the population [1]. Despite extensive research, its pathogenesis is still not fully understood. Primarily, psoriasis is a genetically determined condition and is modified by external environmental factors [2]. In addition, psoriasis is characterized by complex immunological disturbances [3], which lead to reduced apoptosis of keratinocytes, resulting in hyperproliferation and abnormal differentiation. It has been suggested that psoriatic keratinocytes are somehow resistant to apoptosis, while they are susceptible to death by necroptosis [4]. It has been emphasized that keratinocytes are the initiators of the inflammatory cascade, and their death has a significant role in the psoriasis pathogenesis due to the amplification of inflammation [4], which chronically sustained is a feature of psoriatic epidermis. The epidermal turnover time in psoriasis is significantly shortened physiologically from about 28 to only 4 days, which results in the thickening of the epidermis [5]. From a clinical point of view, this translates into infiltrated psoriatic plaques with superficial scaling [5].

Gasdermins are a relatively newly identified family of six proteins, designated A-F, which are engaged in a large number of biological processes, especially associated with cell death, proliferation, differentiation, and migration [6]. With the exception of gasdermin F, they all have a similar chemical structure: a GSDM N-terminal domain, a linker region, and a GSDM C-terminal domain [6]. More precisely, the C-terminal domain plays a controlling and inhibitory role in the activity of the N-terminal domain, which has cytotoxic activity [7]. The specific structure is associated with their ability for autoinhibition and activation, thus resulting in the formation of pores in cellular membranes [8]. Gasdermins can be cleaved by caspases or granzymes leading to the release of an N-terminal fragment, which forms the aforementioned pores in cells causing their death (called pyroptosis) [8]. Different types of caspases may induce such reactions due to various stimuli involved [6,7]. Moreover, the aim of pore formation by gasdermins may vary depending on the target cell, the level of gasdermin protein expression, the timing of activation, and other factors [8].

Gasdermin E (GSDME, previously called DFNA5 protein) is a protein from the gasdermin family, encoded by a gene on chromosome 7p15 [9]. It has an established role in different types of cell death including pyroptosis, apoptosis and necroptosis [7,9]. Directly, GSDME is independent of inflammasome complexes [9]; it is frequently cleaved by caspase 3 but can also be activated by granzyme B [9]. Cleavage of GSDME leads to cell membrane permeabilization and the release of IL-1β and IL-18, but it also leads to the release of pro-apoptotic compounds, cytochrome C and HtrA2, which promote apoptotic protease-activating factor 1 (Apaf1), which further stimulates caspase 3 again [7,9]. Additionally, pores that have been formed can activate the NLRP3 inflammasome, which leads to the maturation of IL-1β [9]. So far, GSDME has been linked, among others, to genetically determined deafness or suppression of malignant tumor growth [7]. It has been also suggested as a marker of prognosis and response to chemotherapy in oral cancer [10].

Considering the established role of GSDME in cell death and proliferation [8], we decided to investigate it as a possible element of psoriasis pathogenesis and potential future marker of psoriasis.

Gasdermins have hardly been studied in psoriasis, and serum and urinary concentrations of gasdermins have never been evaluated in dermatosis before. Our team has already investigated gasdermins A-E in serum, urine and skin samples of psoriatic patients, and we have recently published our findings regarding gasdermin D [11]. Now, we present our outcomes concerning GSDME.

## 2. Materials and Methods

The study enrolled 60 patients in total (21 women, 39 men) with active plaque-type psoriasis, at the mean age of 50 ± 2.34 years old as well as 30 sex- and age-matched volunteers without dermatoses and a negative family history of psoriasis. All participants signed informed consent before enrolment onto the study. The exclusion criteria for the study were as follows: age under 18 years old, pregnancy, types of psoriasis other than plaque, dietary restrictions, intake of oral medications at least 3 months prior to the study, autoimmune diseases other than psoriasis, kidney impairment and malignancies. The severity of psoriasis was assessed using the Psoriasis Area and Severity Index (PASI) and was always performed by the same dermatologist. The study group of patients was divided into three subgroups depending on the severity of the disease: PASI I (PASI < 10)—mild psoriasis; PASI II (PASI 10–20)—moderate psoriasis; and PASI III (PASI > 20)—severe psoriasis. Body mass index (BMI) was calculated as weight/height^2^. Laboratory tests were performed before the study. The study was approved by the Bioethics Committee of the Medical University of Bialystok (APK.002.303.2022 and APK.002.19.2020) and was conducted in accordance with the principles of the Helsinki Declaration [12].

### 2.1. Serum and Urine

The serum and urine analysis was performed in 60 psoriatic patients and 30 sex- and age-matched volunteers without dermatoses. Fasting blood samples were taken using vacuum tubes. They were left to clot for 30 min before centrifugation for 10 min at 2000 *g*. Urine samples were taken as first morning specimens from mid-stream, they were centrifugated for 10 min at 2000 *g*. The obtained serum and urine were stored at −80 °C until further analysis. Laboratory parameters were measured using routine techniques. GSDME concentrations were measured with an enzyme-linked immunosorbent assay (ELISA) provided by EIAab^®^ (Wuhan, China, E2649h) with a minimum detectable dose of 0.156–10 ng/mL. Optical density was read at a wavelength of 450 nm. The concentrations were assessed by interpolation from calibration curves prepared with standard samples provided by the manufacturer. All laboratory tests were performed by the same person in standardized laboratory settings.

### 2.2. Tissue Samples

Tissue sample collection was performed in 33 patients with psoriasis and 20 sex- and age-matched volunteers without skin diseases. All participants did not apply any topical agents on the skin at least a month before the biopsy. Biopsies were taken from the trunk in all participants with a 4 mm punch, after local anesthesia with 2% lignocaine. As for patients, two biopsies were taken: one from the lesional skin—psoriatic plaque—and the other one from the non-lesional, clinically healthy skin—approximately 2 cm from the psoriatic plaque. As for the controls, one skin sample was taken from the non-lesional skin, and the other was performed during the surgical removal of benign skin lesions. Tissue samples were then fixed in 10% buffered formalin solution. After preservation, they were embedded in paraffin blocks and cut into 4 µm sections on silanized slides. The next step was an overnight incubation at 60 °C, and subsequent deparaffinization and rehydration of the tissues. Slides were then incubated with 3% hydrogen peroxide solution to block endogenous peroxidases and protein block to avoid non-specific antibody binding. Next, tissues were incubated with rabbit polyclonal anti-human GSDME antibody (dilution 1:50, Clone OTI1, Origene^®^, Herford, Germany) for 30 min at room temperature. Then, Post Primary Block and Novolink Polymer were used (Leica Novolink Polymer Detection System^®^, Deer Park, IL, USA). Protein expression was visualized with Novolink DAB solution and cell nuclei with hematoxylin (Leica Novolink Polymer Detection System^®^, USA).

**Figure 1:**
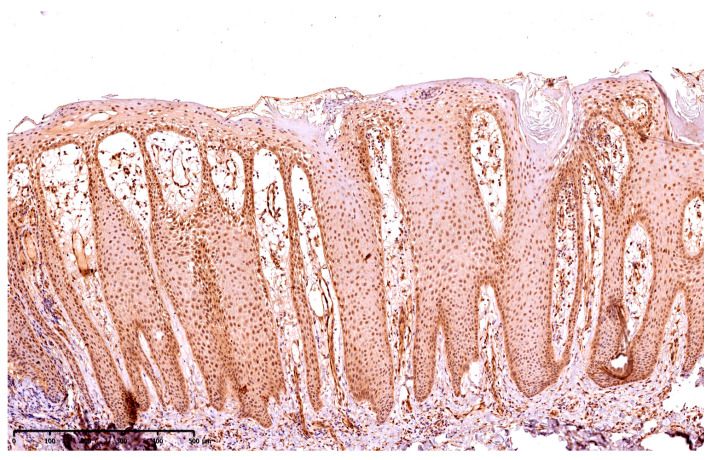
The expression of GSDME in the whole epidermis in the area of the psoriatic plaque; 100× magnification.

**Figure 2:**
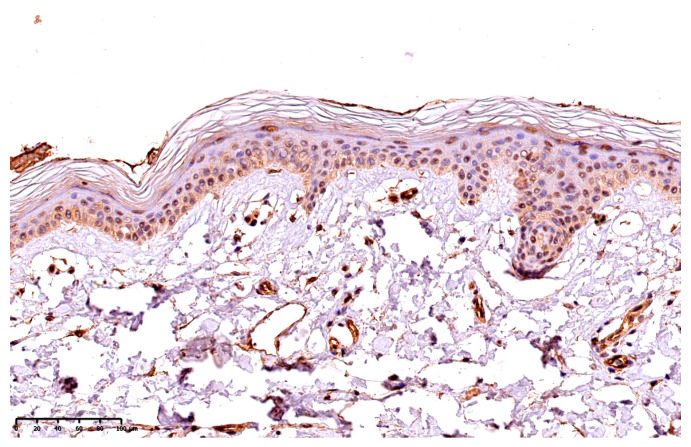
The expression of GSDME in the epidermis of the healthy skin of controls; 200× magnification.

The presence of GSDME was presented using a semi-quantitative method, the reaction was observed on the epidermis as follows: 10—up to 10% keratinocytes from the basement membrane up; 25—up to 25% keratinocytes from the basement membrane up; 50—up to 50% keratinocytes from the basement membrane up; 75—75% keratinocytes from the basement membrane up; 100—in the whole epidermis, except for the stratum corneum. Figure 1 presents the expression of GSDME in the whole epidermis in the area of the psoriatic plaque (Figure 1). Figure 2 presents the expression of GSDME in the epidermis of the healthy skin of controls (Figure 2).

### 2.3. Statistical Analysis

Shapiro–Wilk’s W test of normality was used for data distribution analysis. The normally distributed data were analyzed using the Student’s *t*-test or one-way analysis of variance (ANOVA) and are shown as mean ± SD. The non-Gaussian data were presented as median (full range) and analyzed using the non-parametric the Mann–Whitney test or Kruskal–Wallis test. The relationships between the examined parameters were assessed with the Spearman’s rank test. Statistical analysis was conducted using GraphPad Prism 9.4 software. The differences were deemed statistically significant when *p* < 0.05.

## 3. Results

The basic characteristics of all patients and controls are presented in Table 1.

There was no statistically significant difference between patients and controls in terms of age, gender or BMI.

### 3.1. Serum and Urine

**Figure 3:**
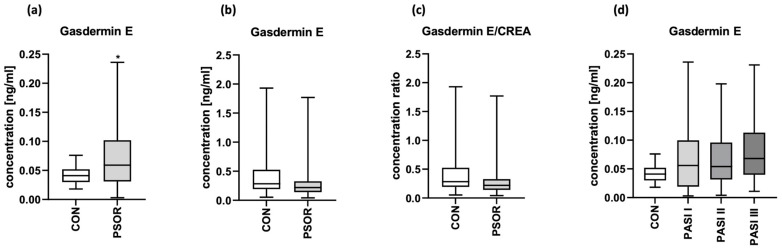
Serum GSDME concentration (**a**), absolute urinary GSDME concentration (**b**) and urinary GSDME/creatinine concentration ratio (**c**) in patients and controls; (**d**) the division of serum GSDME concentrations in patients depending on PASI compared to controls. * means statistically significant difference with *p* < 0.05.

The serum concentration of GSDME was significantly higher in psoriatic patients than in controls (*p* < 0.05) (Figure 3a). Absolute urinary GSDME concentration and the urinary GSDME/creatine concentration ratio were insignificantly lower in patients than controls (Figure 3b,c).

The correlation between serum and urinary GSDME was not significant (*p* > 0.05) (Figure S1).

After the division of patients into three groups depending on psoriasis severity in PASI, median GSDME serum concentrations were higher in patients with the most severe lesions expressed by PASI; however, there was no significant difference between the groups (Figure 3d).

GSDME serum concentrations were higher in women than men; however, they showed no significance. There was no significant correlation between serum concentration of GSDME and PASI, age of patients or psoriasis duration. There was a significant negative correlation between GSDME serum concentration and BMI (R = −0.3; *p* = 0.03).

**Figure 4:**
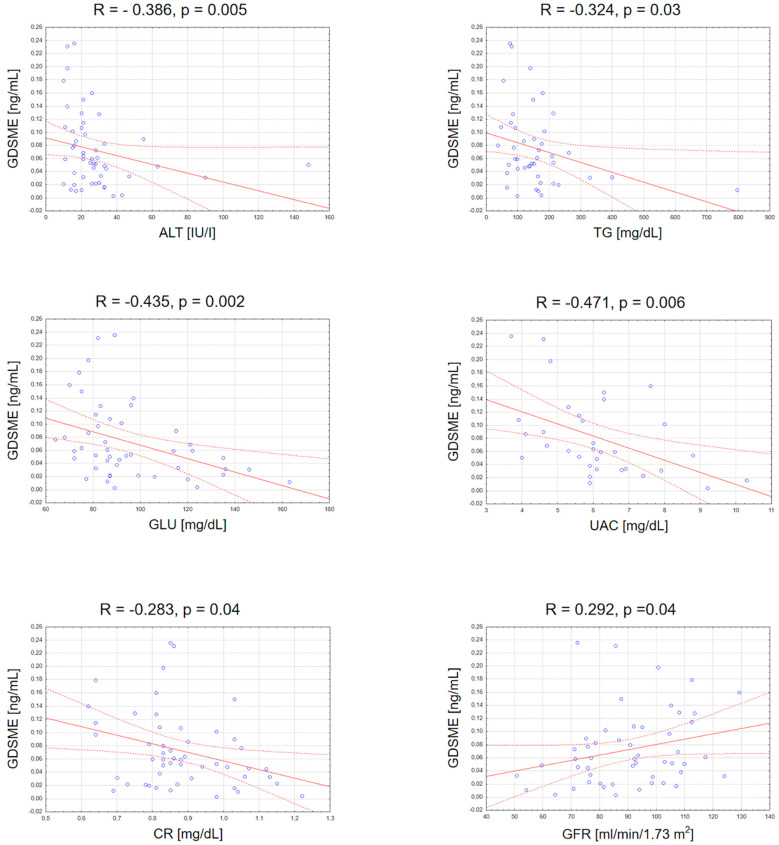
Significant correlations between serum GSDME concentration and basic laboratory parameters. TGs, triglycerides; ALT, alanine transaminase; GLU, fasting glucose; UAC, uric acid; CR, creatinine; GFR, glomerular filtration rate.

There was a negative correlation between GSDME serum concentration and ALT activity (R = −0.39; *p* = 0.005), triglycerides (R = −0.32; *p* = 0.032), glucose (R = −0.43; *p* = 0.002), creatinine (R = −0.04; *p* = 0.44) and uric acid concentrations (R = −0.47; *p* = 0.006), as well as a positive correlation between GSDME and GFR (R = 0.29; *p* = 0.038) (Figure 4). The rest of correlations between GSDME and other laboratory parameters are presented in the supplementary materials (Figure S2).

### 3.2. Tissue

**Figure 5:**
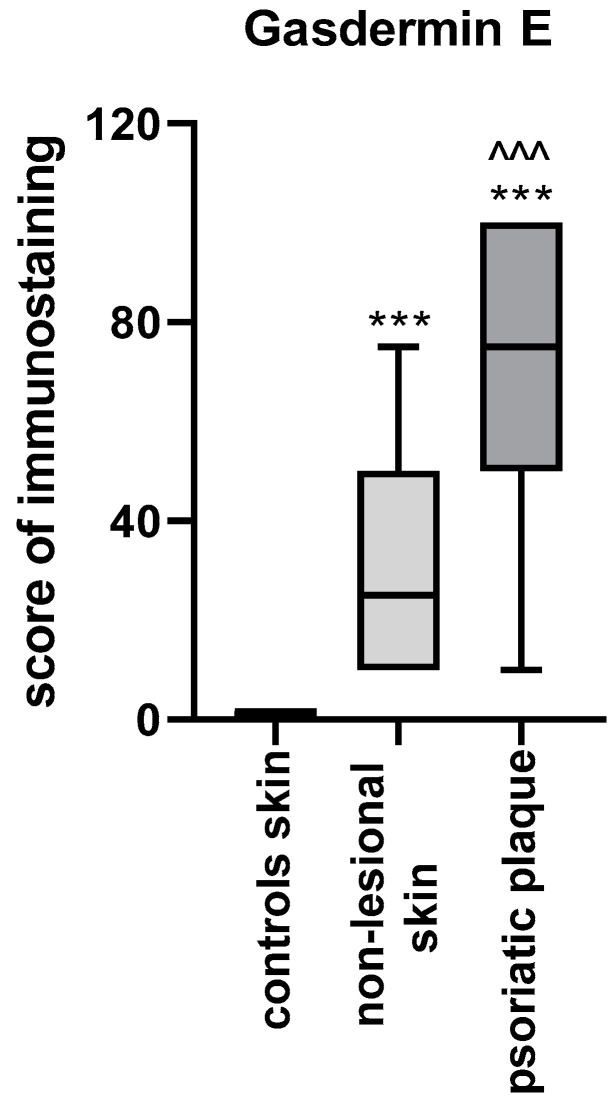
GSDME expression in the skin: psoriatic plaque, non-lesional patients’ skin and healthy controls skin. *** means statistically significant difference with *p* < 0.001 compared to the controls; ^^^ means statistically significant difference with *p* < 0.001 compared to the non-lesional skin.

As for immunohistochemistry, the expression of GSDME was statistically significantly higher in psoriatic plaque tissue (*p* < 0.001) compared with the non-lesional skin in patients and the healthy skin in the control group (*p* < 0.001). The expression in the non-lesional skin was also significantly higher than the healthy skin of controls (*p* < 0.001) (Figure 5).

**Figure 6:**
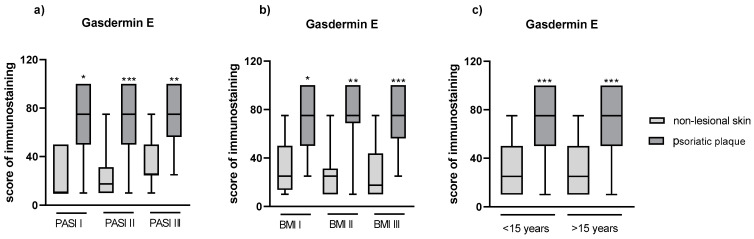
(**a**) The division of GSDME expression in patients depending on PASI, (**b**) BMI, (**c**) psoriasis duration. */**/*** means statistically significant difference with *p* < 0.05/0.01/0.001, respectively, between lesional and non-lesional skin.

After the division into three subgroups according to PASI (Figure 6a) and BMI (Figure 6b), or into two groups according to psoriasis duration (Figure 6c), we did not observe significant differences between GSDME expression in the psoriatic plaque or non-lesional skin between the particular subgroups.

We did not observe any significant correlations between the expression in the psoriatic plaque and PASI, age and duration of psoriasis (*p* > 0.05).

## 4. Discussion

Psoriasis is one of the greatest challenges in contemporary dermatology. Not surprisingly, it has attracted the attention of many scientists. The search for reliable psoriasis markers, markers of its complications and effective therapeutic agents is still ongoing.

Gasdermins have already been somewhat studied in psoriasis but, to the best of our knowledge, there is no research on human serum or urine, which could simultaneously serve as excellent biological materials for patient testing. Experimental studies showed that, besides apoptosis, which disturbances are proven in psoriasis, pyroptosis may actually play a role in this dermatosis as well. It appears that it occurs both in keratinocytes and macrophages [13]. Zhang et al. tried to establish the role of pyroptosis in different dermatoses by assessment of the gasdermins at the single-cell RNAseq level involved in this process in keratinocytes, fibroblasts and macrophages. They stated that the genes are up- or down-regulated depending on the epidermal layer; however, their study was only performed on 13 psoriatic skin samples and 5 controls [13], and so definite conclusions cannot be drawn. The susceptibility for pyroptosis was different among different gasdermins and epidermal layers [13]. As for GSDME, contrary to us, they found a trace expression in all epidermal layers [13]. Besides pyroptosis, GSDME takes part in cell death in the mechanism of apoptosis. It has been proven to exert a suppressive influence on the growth of several neoplasms [9]. For instance, one study reported that under the condition of GSDME overexpression, the proliferation of hepatocellular carcinoma cells was inhibited [9,14], and another study shared similar findings in the case of lung cancer [9,15]. GSDME and other gasdermins have also been previously evaluated with regard to epidermal cornification. Pyroptosis, and thus gasdermins, has been suggested to take part in the process of physiological terminal differentiation in the human epidermis [16]. In a study on human keratinocyte culture using real-time polymerase chain reaction, GSDME was down-regulated during the keratinocyte differentiation process [16]. Our study on psoriatic tissue, which exhibits abnormal epidermal differentiation, revealed higher GSDME expression compared to healthy skin.

GSDME can be cleaved by granzyme B or caspase 3, but due to the feedback, it can also indirectly additionally lead to the cleavage of gasdermin D (GSDMD), which is followed by pyroptosis and inflammatory cytokines release [7]. Nowadays, it is believed that in case of cell death mediated by GSDME, pyroptosis is tightly associated with apoptosis via caspase 3 [9].

GSDME has been linked to inflammatory diseases. GSDME-mediated pyroptosis was suggested as a mechanism in periodontonitis [9,17], rheumatoid arthritis [9,18] and inflammatory bowel diseases [9,19], which are more frequent in psoriatic patients [20]. GSDME has also been documented to participate in skin inflammation. Liu et al. conducted an experiment on a model of keratinocytes-mediated inflammation induced by UVB radiation. They proved pyroptosis occurrence and revealed up-regulation of GSDME as the mediator in this process, leading to skin inflammation [21]. Similar observations were first made by Chen et al. [22]. The same team extended their research and found later that after UVB radiation exposure, different cell deaths in the mice’s epidermis occurred at the same time, namely GSDME-mediated pyroptosis, apoptosis and necroptosis [23], which confirms the diverse nature of GSDME we mentioned in the introduction. GSDME has also been reported to play a role as a mediator in the sustained chronic inflammatory skin condition in zebra fish [24].

We obtained meaningful results—GSDME concentration was significantly higher in the serum of patients, and its expression was significantly higher in psoriatic lesions than in non-lesional skin of patients or the healthy skin of subjects without dermatoses. This could indicate the involvement of GSDME in psoriasis.

It has been suggested that GSDME may direct cells towards pyroptosis or apoptosis depending on its own expression. Apoptosis is presumed to occur when the expression of GSDME is low, and pyroptosis occurs when it is highly expressed [9]; however, this process does not appear to be that simple. Given the higher expression of GSDME in psoriatic tissue compared to non-lesional skin, it either means that the cells are going to be subject to pyroptosis, which leads to a sustained inflammatory condition and explains why apoptosis is decreased, or GSDME is compensatively elevated to direct the cells towards apoptosis. Considering the following: GSDME is able to direct cells towards apoptosis, psoriasis is known to decrease apoptosis, and finally, GSDME expression in psoriatic lesions and concentration in serum in our study is increased, we could assume it is indeed compensatively elevated. Perhaps, it could be explained in such a way that GSDME could play a protective role in individuals with psoriasis counteracting keratinocytes’ resistance to apoptosis. Nevertheless, considering that GSDME apoptosis is related to pyroptosis and the feedback mechanism of GSDMD cleavage due to caspase 3, it is possible the two processes may coexist and additional activation of GSDMD further exacerbates inflammation. It has already been raised by Liao et al. that however beneficial GSDME is as a pro-apoptotic agent in pathological cells, GSDME is also engaged in disease development via sustained inflammation [9]. Therefore, GSDME could be a multi-faceted protein, and future studies are needed to further examine its role in psoriasis.

Considering the GSDME higher lesional expression and serum concentration in psoriatics, GSDME could become a new marker of psoriasis, especially its serum—a relatively non-invasive one. At the same time, our results do not allow us to assume that GSDME could serve as a marker of psoriasis severity because there were no correlations or significant differences in its concentrations or expression depending on PASI. However, in subjects with the most severe lesions, serum GSDME concentration was higher, and so it requires more in-depth studies on larger samples to assess this matter. Obviously, we must take into account that we used PASI for the assessment of skin lesions, and, however popular, it is a subjective scoring method; hence, the results may differ between assessing dermatologists. We also do not have any similar data from other studies to discuss ours with.

Serum concentration and lesional expression of GSDME were independent of gender and psoriasis duration; therefore, it seems to be potentially useful equally in both sexes, but probably could not serve as markers of the time of suffering from this dermatosis.

A very important issue seems to be the emerging role of GSDME in terms of metabolic disorders. Our results show a negative correlation between GSDME serum concentration and BMI, ALT activity, triglycerides, glucose and uric acid concentrations, whose elevated levels are associated with the components of metabolic syndrome (MS), pointing to its potential role as a protective agent in these conditions. Unfortunately, there are no data on this matter in the available literature. To the best of our knowledge, GSDME has never been investigated in relation to metabolic complications, much less in psoriasis. At the same time, psoriasis is well-documented to be associated with MS, and psoriatics live approximately 5 years shorter than subjects without psoriasis due to cardiovascular incidents [25]. Therefore, there are even guidelines on screening psoriatics for metabolic complications [26]. The development of a potential marker indicating increased risk in this group of patients would be of great value.

Part of our investigation was to examine urine as a body fluid that could potentially be useful for the evaluation of gasdermins. The biggest advantage of urine collection is that it is non-invasive and painless, and more patients are happy approve of its donation. These are essential issues from the perspective of daily clinical practice and scientific research. On the other hand, the main disadvantage of urine analysis is the difference in kidney function, usually expressed by GFR, which can affect the concentrations of gasdermins in urine. This is why we presented our results both as absolute values of urine GSDME concentrations and as the ratio to urine creatinine concentration. Nevertheless, in both cases, the differences between patients and controls were not significant; hence, urine is unlikely to become useful as a fluid for GSDME assessment, but future studies are welcome to investigate this issue. We are not aware of any other studies evaluating gasdermins in urine.

The limitation of our study is the relatively low number of participants of one ethnicity. On the other hand, we know from experience that patients are usually not willing to donate skin samples for research, which makes tissue studies more difficult than those based on blood examination. Moreover, the limited data on the kidney metabolism of gasdermins makes urine results difficult to interpret. Finally, more studies on bigger samples are required to deeper explore this issue.

## 5. Conclusions

We are the first to report the possible serum and urine GSDME applications in psoriatics. We proved that GSDME serum concentration, its expression in psoriatic plaques and even in non-lesional skin is significantly elevated in patients with psoriasis compared to controls without psoriasis. Based on our results and accounting for the biological function of gasdermins, especially cell death, it is reasonable to suspect they could participate in psoriasis pathogenesis. Serum GSDME could be further analyzed as a potential non-invasive marker of psoriasis and an interesting target for anti-psoriatic drugs. On the other hand, GSDME does not currently seem to be a marker of psoriasis severity. A crucial matter that requires more in-depth studies is the role of GSDME as a protective agent against metabolic complications in psoriasis.

## 6. Patents

Serum GSDME is the subject of an ongoing patent application no. P.445837.

## Figures and Tables

**Table 1:** Basic characteristics of patients and controls.

Parameter	Controls (n = 30)	Psoriatic Patients (n = 60)
Sex (M/F)	20/10	39/21 NS
Age (years)	48 ± 2.45	50 ± 2.34 NS
Height (cm)	1.75 (1.5–1.9)	1.71 (1.5–1.9) NS
Weight (kg)	78.40 ± 2.9	85.43 ± 2.5 NS
BMI	25.85 ± 0.77	27.85 ± 0.64 NS

NS, non-significant difference.

## Data Availability

Data available upon request from the authors.

## Supplementary Materials

The following supporting information can be downloaded at: https://www.mdpi.com/article/10.3390/cells12172149/s1, Figure S1. The correlation between serum and urinary GDSME. Figure S2. Correlations between GSDME and other laboratory parameters.

## References

[1] Grän F., Kerstan A., Serfling E., Goebeler M., Muhammad K. (2020). Current Developments in the Immunology of Psoriasis. Yale J. Biol. Med..

[2] Hirotsu C., Rydlewski M., Araújo M.S., Tufik S., Andersen M.L. (2012). Sleep loss and cytokines levels in an experimental model of psoriasis. PLoS ONE.

[3] Lanna C., Mancini M., Gaziano R., Cannizzaro M.V., Galluzzo M., Talamonti M., Rovella V., Annicchiarico-Petruzzelli M., Melino G., Wang Y. (2019). Skin immunity and its dysregulation in psoriasis. Cell Cycle.

[4] Shou Y., Yang L., Yang Y., Xu J. (2021). Inhibition of keratinocyte ferroptosis suppresses psoriatic inflammation. Cell Death Dis..

[5] Lowes M.A., Suárez-Fariñas M., Krueger J.G. (2014). Immunology of psoriasis. Annu. Rev. Immunol..

[6] Kovacs S.B., Miao E.A. (2017). Gasdermins: Effectors of Pyroptosis. Trends Cell Biol..

[7] Kuc-Ciepluch D., Ciepluch K., Arabski M. (2021). Gasdermin family proteins as a permeabilization factor of cell membrane in pyroptosis process. Postep. Hig. Med. Dosw..

[8] Zou J., Zheng Y., Huang Y., Tang D., Kang R., Chen R. (2021). The Versatile Gasdermin Family: Their Function and Roles in Diseases. Front. Immunol..

[9] Liao X.X., Dai Y.Z., Zhao Y.Z., Nie K. (2022). Gasdermin E: A Prospective Target for Therapy of Diseases. Front. Pharmacol..

[10] Wang S., Zhang M.J., Wu Z.Z., Zhu S.W., Wan S.C., Zhang B.X., Yang Q.C., Xiao Y., Chen L., Sun Z.J. (2022). GSDME Is Related to Prognosis and Response to Chemotherapy in Oral Cancer. J. Dent. Res..

[11] Nowowiejska J., Baran A., Hermanowicz J.M., Pryczynicz A., Sieklucka B., Pawlak D., Flisiak I. (2023). Gasdermin D (GSDMD) Is Upregulated in Psoriatic Skin—A New Potential Link in the Pathogenesis of Psoriasis. Int. J. Mol. Sci..

[12] Helsinki Declaration. https://www.wma.net/policies-post/wma-declaration-of-helsinki-ethical-principles-for-medical-research-involving-human-subjects/.

[13] Zhang D., Li Y., Du C., Sang L., Liu L., Li Y., Wang F., Fan W., Tang P., Zhang S. (2022). Evidence of pyroptosis and ferroptosis extensively involved in autoimmune diseases at the single-cell transcriptome level. J. Transl. Med..

[14] Wang C.J., Tang L., Shen D.W., Wang C., Yuan Q.Y., Gao W., Wang Y.K., Xu R.H., Zhang H. (2013). The Expression and Regulation of DFNA5 in Human Hepatocellular Carcinoma DFNA5 in Hepatocellular Carcinoma. Mol. Biol. Rep..

[15] Kong Y.H. (2013). The Biological Function and Deaf Mechanism of DFNA5. Master’s Thesis.

[16] Lachner J., Mlitz V., Tschachler E., Eckhart L. (2017). Epidermal cornification is preceded by the expression of a keratinocyte-specific set of pyroptosis-related genes. Sci. Rep..

[17] Liu J., Wang Y., Meng H., Yu J., Lu H., Li W., Lu R., Zhao Y., Li Q., Su L. (2019). Butyrate rather Than LPS Subverts Gingival Epithelial Homeostasis by Downregulation of Intercellular Junctions and Triggering Pyroptosis. J. Clin. Periodontol..

[18] Zhai Z., Yang F., Xu W., Han J., Luo G., Li Y., Zhuang J., Jie H., Li X., Shi X. (2022). Attenuation of Rheumatoid Arthritis through the Inhibition of Tumor Necrosis Factor-Induced Caspase 3/Gasdermin E-Mediated Pyroptosis. Arthritis Rheumatol..

[19] Xu Z., Liu R., Huang L., Xu Y., Su M., Chen J., Geng L., Xu W., Gong S. (2020). CD147 Aggravated Inflammatory Bowel Disease by Triggering NF-Κb-Mediated Pyroptosis. Biomed. Res. Int..

[20] Fu Y., Lee C.H., Chi C.C. (2018). Association of Psoriasis with Inflammatory Bowel Disease: A Systematic Review and Meta-analysis. JAMA Dermatol..

[21] Liu J., Zhong Y., Liu H., Yang H., Lu P., Shi Y., Wang X., Zheng W., Yu X., Xu Y. (2021). Oncostatin M sensitizes keratinocytes to UVB-induced inflammation via GSDME-mediated pyroptosis. J. Dermatol. Sci..

[22] Chen Y., Xiao T., Xu S., Gu H., Li M., Chen X. (2020). Ultraviolet B induces proteolytic cleavage of the pyroptosis inducer gasdermin E in keratinocytes. J. Dermatol. Sci..

[23] Chen Y., Lian N., Chen S., Xiao T., Ke Y., Zhang Y., Song C., Yang Y., Xu S., Gu H. (2022). GSDME deficiency leads to the aggravation of UVB-induced skin inflammation through enhancing recruitment and activation of neutrophils. Cell Death Dis..

[24] Lozano-Gil J.M., Rodríguez-Ruiz L., Tyrkalska S.D., García-Moreno D., Pérez-Oliva A.B., Mulero V. (2022). Gasdermin E mediates pyroptotic cell death of neutrophils and macrophages in a zebrafish model of chronic skin inflammation. Dev. Comp. Immunol..

[25] Ryan C., Kirby B. (2015). Psoriasis Is a Systemic Disease with Multiple Cardiovascular and Metabolic Comorbidities. Dermatol. Clin..

[26] Elmets C.A., Leonardi C.L., Davis D.M., Gelfand J.M., Lichten J., Mehta N.N., Armstrong A.W., Connor C., Cordoro K.M., Elewski B.E. (2019). Joint AAD-NPF guidelines of care for the management and treatment of psoriasis with awareness and attention to comorbidities. J. Am. Acad. Dermatol..

