# Supplementary material for: Gasdermin E (GSDME)—A New Potential Marker of Psoriasis and Its Metabolic Complications: The First Combined Study on Human Serum, Urine and Tissue
Source: Cells. 2023 Aug 26;12(17):2149. doi: 10.3390/cells12172149 (PMC10486754; doi:10.3390/cells12172149)
Supplement: Supplementary file 1 [file cells-12-02149-s001.zip › cells-2543611-supplementary.pdf]

Supplementary files

Figure S1. The correlation between serum and urinary GSDME.

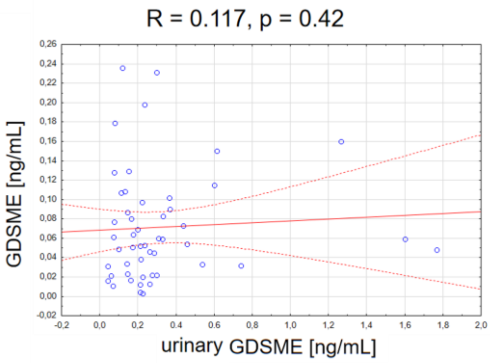

Figure S2. Correlations between GSDME and other laboratory parameters: (a) WBC, (b) RBC, (c) HGB, (d) AST, (e) PLT, (f) total cholesterol, (g) HDL, (h) LDL, (i) CRP, (j) urea, (k) BUN.
WBC, white blood cells; RBC, red blood cells; HGB, hemoglobin; AST, asparagine transaminase; PLT, platelets; Chol, total cholesterol; HDL, high-density lipoprotein; LDL, low-density lipoprotein; CRP, C-reactive protein; BUN, blood urea nitrogen; UAC, uric acid.

(a)

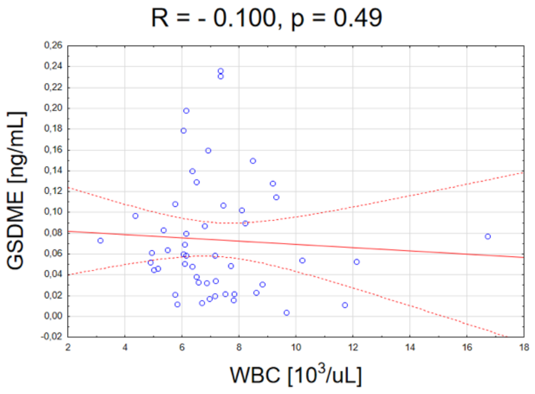

(b)

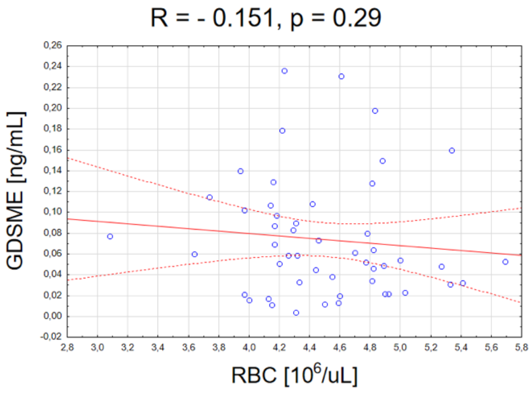

(c)

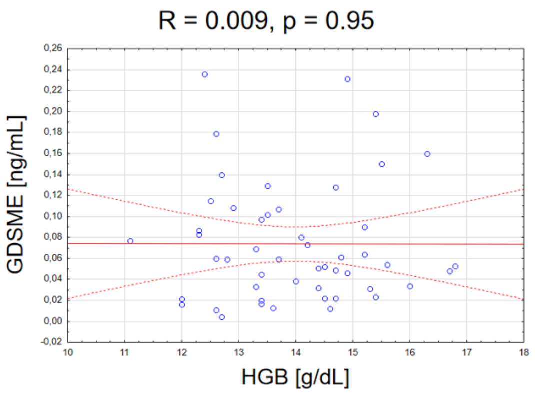

(d)

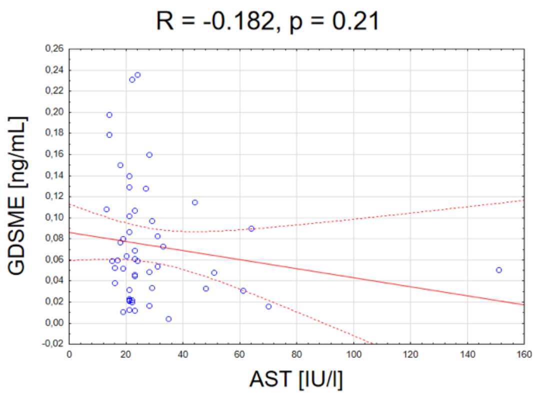

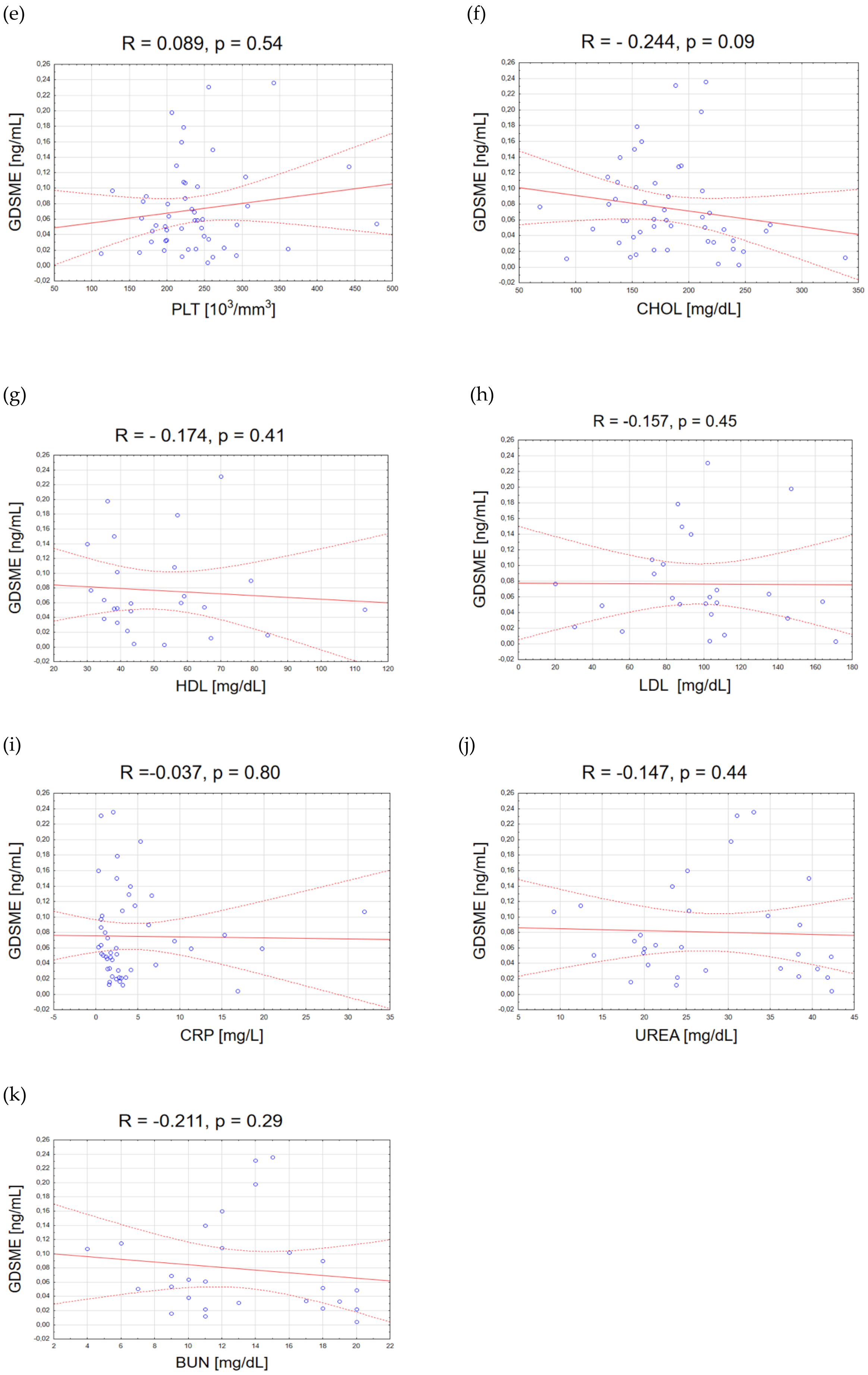

(e)
R = 0.089, p = 0.54
GDSME [ng/mL]
PLT [10^3/mm^3]
(f)
R = - 0.244, p = 0.09
GDSME [ng/mL]
CHOL [mg/dL]
(g)
R = - 0.174, p = 0.41
GDSME [ng/mL]
HDL [mg/dL]
(h)
R = -0.157, p = 0.45
GDSME [ng/mL]
LDL [mg/dL]
(i)
R =-0.037, p = 0.80
GDSME [ng/mL]
CRP [mg/L]
(j)
R = -0.147, p = 0.44
GDSME [ng/mL]
UREA [mg/dL]
(k)
R = -0.211, p = 0.29
GDSME [ng/mL]
BUN [mg/dL]
